# Green Synthesis, Characterization, Antimicrobial and Anticancer Screening of New Metal Complexes Incorporating Schiff Base

**DOI:** 10.1021/acsomega.2c03911

**Published:** 2022-08-26

**Authors:** Ali M. Hassan, Ahmed O. Said, Bassem H. Heakal, Ahmed Younis, Wael M. Aboulthana, Mohamed F. Mady

**Affiliations:** † Chemistry Department, Faculty of Science, Al-Azhar University, Nasr City 11884, Egypt; ‡ Senior researcher chemist, Greater Cairo Water Company, Cairo 11047, Egypt; § Research Laboratory, Cairo Oil Refining Company, Mostorod 11757, Kaliobia, Egypt; ∥ Department of Green Chemistry, National Research Centre, Cairo 12622, Egypt; ⊥ Biochemistry Department, Biotechnology Research Institute, National Research Centre, Dokki, 12622 Giza, Egypt; # Department of Chemistry, Bioscience and Environmental Engineering, Faculty of Science and Technology, University of Stavanger, N-4036 Stavanger, Norway

## Abstract

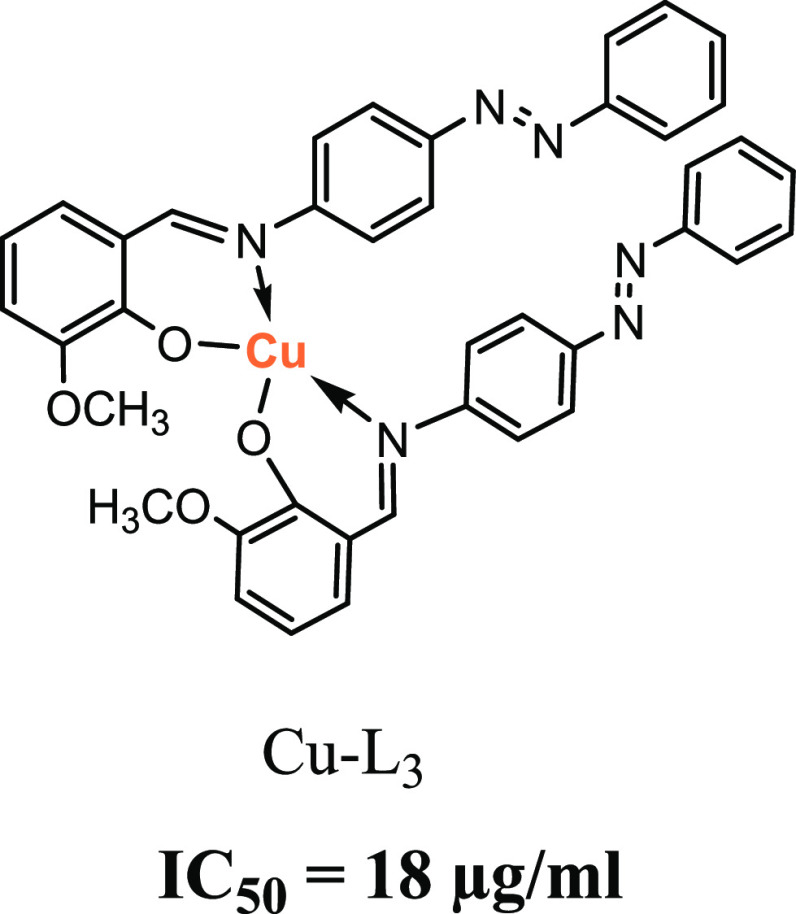

A Schiff base ligand of *o*-vanillin and 4-aminoazobenzene and its transition metal complexes of Ni(II), Co(II), Zn(II), Cu(II), Mn(II), and Zr(IV) were prepared under microwave irradiation as a green approach compared to the conventional method. The structures of new compounds have been characterized and elucidated via elemental and spectroscopic analyses. In addition, magnetic susceptibility, electron spin resonance, and electronic spectra of the synthesized complexes explained their geometrical structures. The thermal stability of Cu(II), Zn(II), and Zr(IV) complexes was studied by thermo-gravimetric analyses (TGA). Coats–Redfern and Horowitz–Metzger equations were used to calculate the thermal and dehydration decomposition activities of proposed structures kinetically. Surface morphologies of the solid compounds were imaged by scanning electron microscopy (SEM). The particle size of prepared complexes was measured by using a particle size analyzer at a diffraction angle of 10.9°. The geometry structures of the synthesized compounds were verified utilizing electronic spectra, ESR spectrum, and magnetic moment value. The newly synthesized compounds were screened for antimicrobial activity. Also, the anticancer activity of the free Schiff base ligand and its metal complexes were studied against two cell lines: human colon (HCT-116) and human liver cancer cells (HepG-2). The obtained results showed that the Cu(II) complex displayed the highest cytotoxic activity (IC_50_ = 18 and 22 μg/mL for HepG-2 and HCT, respectively) compared to the free Schiff base ligand.

## 1 Introduction

One of the most important parts of coordination chemistry is designing ligands such as Schiff bases with bidentate or multi-dentate behavior from several amines and aldehydes and their coordination with various transition metal ions to form new complexes.^1−9^ Schiff bases derived from *o*-vanillin and salicylaldehyde are widely used as bidentate or multi-dentate ligands because of the presence of a hydroxyl group in the ortho position beside the azomethine group, which formed from binding of amine and aldehyde groups. Schiff bases and their metal complexes have varied applications in solar cell, catalysis, biosensing, antibacterial, antifungal, antiviral, and antitumor.^10−15^ Azomethines are widely employed in other industrial applications, such as paint and dye manufacturing, organic semiconductors, polymers, and corrosion inhibitors.^16,17^*Ortho*-vanillin is also the greatest prominent principal flavor and odor compound in a vanilla plant, which is used as food flavor, in drinks, and in pharmaceuticals.^18^ Because of its many biological activities, it is extensively studied in healing fields.^19−21^

Microwave-assisted synthesis is considered one of the most crucial subdivisions of green chemistry.^22−25^ Microwave reactions under free or less solvent conditions are eye-catching and present reduced pollution, low price, and high productivity together with ease in processing and handling.^26−28^ In recent decades, microwave heating has been generally used in organic and organometallic synthesis and metal–organic frameworks (MOFs) in a wide range.^29−31^ In continuation of our work in the field of utilization of different green chemistry tools in the synthesis of new compounds, in this study, we used the microwave technique to synthesize a Schiff base ligand from the condensation of an equimolar amount of *o*-vanillin and 4-aminoazobenzene and its new metal complexes (1–6) compared to the traditional method. In addition, the *ortho*-vanillin Schiff bases were chosen because their compounds have the ability to interact with DNA and act as anti-bactrial , anti-fungal , anti-cancer, and antioxidant and have many other applications such as being catalysts, polymers, and dyes.^32^ In our work, we screened these compounds against various cancer cells and microbial strains.

## 2 Materials and Methods

### 2.1 Materials

*Ortho*-vanillin and 4-aminoazobenzene were obtained from Sigma Aldrich. All solvents and chemicals were of annular AR grade and used as received. All metal salts were purchased from ADWIC except zirconyl oxychloride, which was purchased from Acros Organics. Microwave-assisted synthesis reactions were carried out in-house using a modified domestic microwave. The purity of the Schiff base ligand and its complexes were detected by using the thin layer chromatography (TLC) technique. Metal contents were determined by complexometric titration using xylenol orange (XO) as an indicator and hexamine as a buffer (pH = 6). Melting points were recorded in open capillaries with a Barnstead Thermolyne Mel-temp 1001D Electrothermal Melting Point.

Elemental analysis was done on a Perkin Elmer PE 2400 CHN elemental analyzer, and a mass spectrometric spectrum for the Schiff base ligand was carried out using a direct inlet unit (DI-50) in the Shimadzu QP-5050 GC–MS at the Regional Center for Mycology and Biotechnology, Al-Azhar University. The FTIR spectra samples were ground with potassium bromide (KBr) powder and then pressed into a disk and recorded on a Shimadzu FTIR-340 Jasco spectrophotometer at the micro-analytical center at Cairo University.^32^ The UV–vis range (9090–52,631 cm^–1^) using a Jenway 6715 UV/Vis spectrophotometer and the morphology of the complexes were examined using JEOL-JSM-6390 OLA Analytical scanning electron microscopy (SEM) at a holding company for water and wastewater.

^1^H NMR spectra for the Schiff base ligand were recorded in dimethyl sulfoxide (DMSO) solution using Bruker’s high-performance Avance III NMR spectrometer 400 MHz, and thermal gravimetric analysis measurements (TGA) were carried out with a Shimadzu thermal analyzer model 50 at the Micro Analytical Center, Cairo University. The ESR spectra of the powdered Cu(II) complex were recorded at room temperature by an X-band EMX spectrometer (Bruker, Germany) using a standard rectangular cavity of ER 4102 with 100 kHz frequency at the National Center for Radiation Research and Technology, Egyptian Atomic Energy Authority. The particle size distribution of metal complexes was determined by laser light scattering on a Beckman coulter particle size analyzer (n5 submicron particle size analyzer, Japan) at the City of Scientific Research and Technological Applications, Alexandria, Egypt. The thermal kinetic parameters of complex decomposition, mostly enthalpy (ΔH*), activation energy (E*), entropy (ΔS*), and Gibbs free energy change of the decomposition (ΔG*), were calculated by two methods (Hawetezmetzegar and Coats Redfern).

### 2.2 Synthesis of Free Schiff Base Ligand (OV-Azo)

#### 2.2.1 Conventional Method

A total of 1.97 g of 4-aminoazobenzene (10 mmol) was dissolved in 30 mL of absolute methanol and mixed with 30 mL of methanolic solution of *ortho*-vanillin (1.52 g, 10 mmol). The mixture was heated and stirred under reflux. The reaction was monitored by TLC and completed after 120 min. The solution was left to cool and precipitate at room temperature. The precipitated crude product was then recrystallized from ethanol to afford an orange precipitate of the Schiff base ligand in a 93% yield.

#### 2.2.2 Microwave Method

An equimolar amount of 4-aminoazobenzene (0.197 g, 1 mmol) and *ortho*-vanillin (0.152 g, 1 mmol) was mixed thoroughly in a mortar and pestle. A few drops of ethanol were added to the reaction mixture. The reaction mixture was subjected to the microwave oven at 360 W. The reaction was completed after 10 min (monitored by TLC). The products were recrystallized with hot ethanol and finally dried under reduced pressure over anhydrous CaCl_2_ in a desiccator to afford a Schiff base ligand in 94% yield. The prepared Schiff base ligand was characterized by spectroscopic techniques FTIR, NMR, and elemental analysis.

2-Methoxy-6-((*E*)-((4-((*E*)-phenyldiazenyl)phenyl)imino)methyl)phenol*.*^33−35,43^ IR ν max (cm^–1^): 1612 (C=N), 1490 (N=N),1261 (C–O methoxy), 3043 (C–H, aromatic), 2962 (C-H, aliphatic), and 3421 cm^–1^ (OH), ^1^H NMR (DMSO-*d*_6_, δ, 400 MHz): δ ppm: 12.94 (s, 1H, OH), 9.04 (s, 1H, azomethine CH=N), 6.91–8.00 (m, 12H, Ar–H), 3.84 (s, 3H, O–CH_3_), elemental analysis: C% (found = 73.2, calcd = 72.49), H% (found = 5.42, calcd = 5.17), N% (found = 13.1, calcd = 12.68), anal. calcd for (C_20_H_17_O_2_N_3_, M. Wt = 313.37).

### 2.3 Synthesis of OV-Azo Metal Complexes

#### 2.3.1 Conventional Method

A total of 10 mmol of hydrated metal acetate (M(CH_3_CO_2_)_2_)·*n*H_2_O, where M = Mn(II), Co(II), Ni(II), Cu(II), and Zn(II)), was dissolved in an appropriate amount of methanol, or 10 mmol of Zr(IV) oxychloride octahydrate was added to a methanolic solution of the Schiff base ligand (OV-Azo) (3.31 g, 10 mmol). The mixture was heated and stirred under reflux for an appropriate time (Table1), left standing overnight. The precipitated product was filtered, washed with ethanol then with diethyl ether, and recrystallized from hot ethanol to afford the desired product.

**Table 1:** Melting Points, Yields, Reaction Time, and Analytical and Physical Properties of the Ligand and Its Complexes

		conventional		microwave				elemental analysis found/(calcd) %				
molecular formula	symbol	time	yield	time	yield	M.P. °C	color	C	H	N	M	M^+^ found (calcd)%
C_20_H_17_O_2_N_3_	OV-Azo L	120 min	93%	10 min	94%	138	orange	73.2 (72.49)	5.42 (5.17)	13. 1 (12.68)		331.2 (331.37)
C_24_H_26_O_8_N_3_ Mn	Mn-L (1)	60 min	80%	12 min	91%	141	dark khaki	53.84 (53.44)	5.01 (4.86)	6.94 (7.79)	10.45 (10.18)	(539.42)
C_24_H_26_O_8_N_3_ Co	Co-L (2)	75 min	83%	9 min	94%	130	brown	53.68 (53.05)	4.77 (4.82)	5.52 (7.73)	11.2 (10.85)	(543.41)
C_24_H_36_O_13_N_3_ Ni	Ni-L (3)	60 min	81%	11 min	91%	134	brown	45.18 (45.52)	5.29 (5.73)	5.45 (6.64)	9.37 (9.27)	(633.25)
C_40_H_33_O_4_N_6_ Cu	Cu-L (4)	60 min	69%	8 min	92%	205	dark brown	65.32 (66.33)	4.67 (4.45)	9.58 (11.6)	9.17 (8.77)	(724.28)
C_60_H_50_O_6_N_9_ Zn	Zn-L (5)	75 min	65%	10 min	86%	254	orange	69.52 (68.15)	4.20 (4.67)	10.38 (11.92)	6.65 (6.18)	(1057.49)
C_20_H_36_O_13_N_3_Cl_2_ Zr	Zr-L (6)	75 min	75%	8 min	92%	200	maroon	34.92 (34.88)	4.24 (5.27)	4.84 (6.1)	13.15 (13.25)	688.91 (688.64)

#### 2.3.2 Microwave Method

An equimolar amount of Schiff base ligand (OV-Azo) (1.655 g, 5 mmol) and hydrated metal acetate or Zr(IV) oxychloride octahydrate (5 mmol) were ground in a mortar and pestle. A few drops of ethanol were added to the reaction mixture. The reaction mixture was subjected to the microwave oven for the appropriate time, as shown in Table 1. The products were recrystallized with hot ethanol and finally dried under reduced pressure over anhydrous CaCl_2_ in a desiccator. The progress of the reaction and purity of the product was monitored by the TLC technique. The reactions were completed in short times with higher yields compared to the conventional method (Table 1). The physical, analytical, and spectra data are given in Tables 1–3.

### 2.4 Antimicrobial Activity

The obtained metal complexes were screened for their biological activities as antibacterial agents against Gram-positive *Staphylococcus aureus* (ATCC 25923) and *Bacillus subtilis* (ATCC 6635); Gram-negative species of *Escherichia coli* (ATCC 25922) and *Salmonella typhimurium* (ATCC 14028), anti-fungal *Candida albicans* (ATCC 10231), and fungus *Aspergillus fumigatus*. Antimicrobial activity was tested by the disc diffusion method.^36^ The cephalothin, chloramphencol, and cycloheximide were used as standard references for Gram-positive and Gram-negative bacteria and fungi, respectively, serving as positive controls. Nutrient agar was then prepared autoclaved at 121 °C for 15 min, cooled, and finally poured into Petri dishes. The tested compounds were dissolved in dimethyl sulfoxide (DMSO) solvent and prepared in two concentrations; 100 and 50 mg/mL and then 10 μL of each preparation was dropped on a disk of 6 mm in diameter, and the concentrations became 1 and 0.5 mg/disk, respectively. Bacterial cultures were grown in a nutrient broth medium at 30 °C. After 16 h of growth, each microorganism, at a concentration of 108 cells/mL, the tested compounds were inoculated on the surface of Mueller–Hinton agar plates using a sterile cotton swab. Subsequently, uniform size filter paper disks (6 mm in diameter) were impregnated by an equal volume (10 μL) from the specific concentration of dissolved compounds and carefully placed on the surface of each inoculated plate. The plates were incubated in the upright position at 36 °C for 24 h. Three replicates were carried out for each extract against each test organism. Simultaneously, the addition of the respective solvent instead of the dissolved compound was carried out as negative control. After incubation, the diameters of the growth inhibition zones formed around the disc were measured with a transparent ruler in millimeters and averaged, and the mean values are recorded in Table 6.

### 2.5 Cytotoxic Evaluation

Cytotoxic activity test (in vitro bioassay on human tumor cell lines) was conducted and determined. It was performed on a human hepatocellular carcinoma cell line (HepG-2) based on the method reported by Mosmann.^37^ and a human colon carcinoma cell line according to the protocol suggested by Vichai and Kirtikara.^38^ All the tumor cells were purchased from CSIR-National Chemical Laboratory, Pune, India. An MTT colorimetric assay was used to plot a dose–response curve required to kill 50% of the cell population (IC_50_). The results are shown in Table 7.

## 3 Results and Discussion

**Scheme 1:**
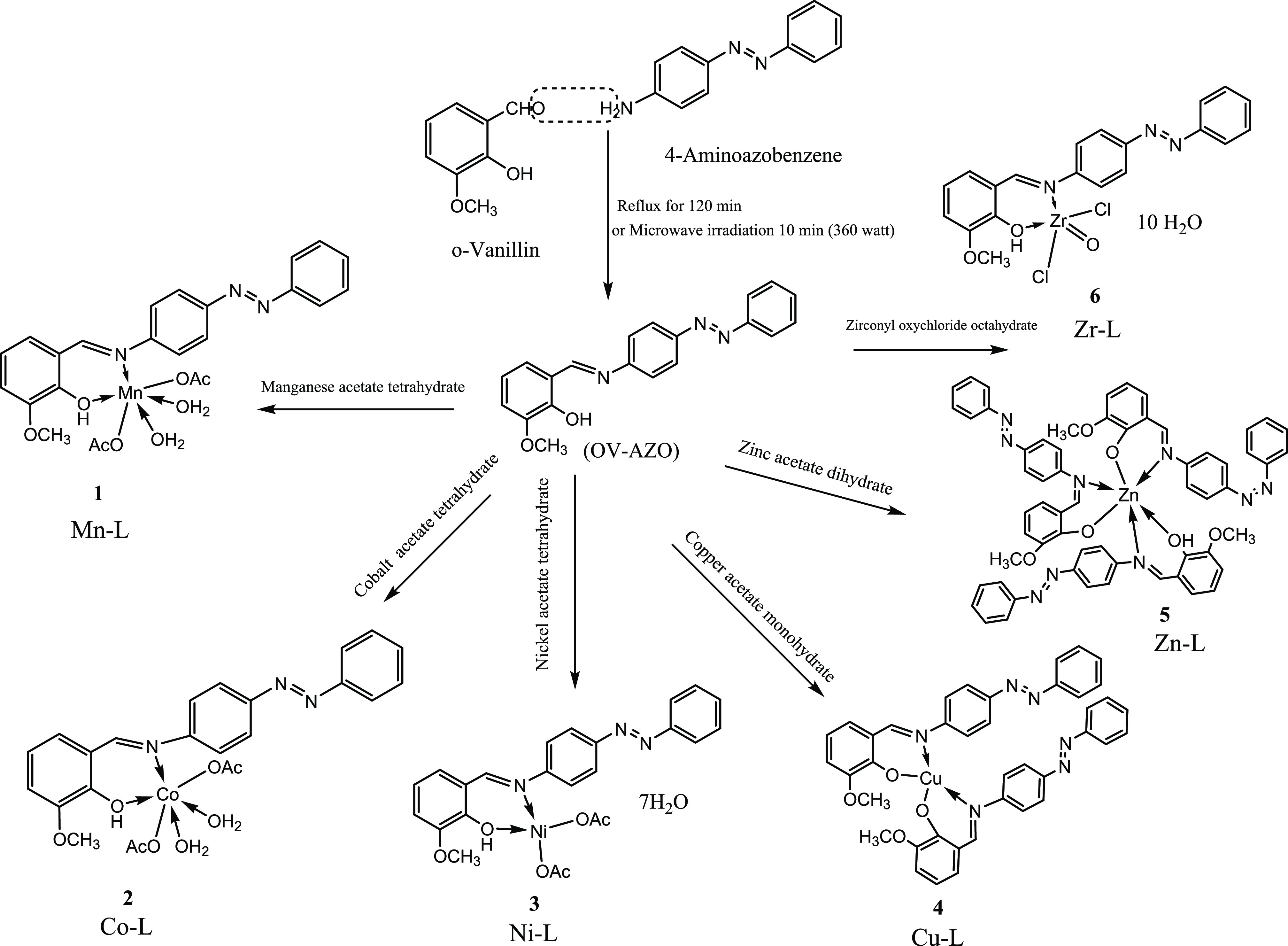
Proposed Molecular Structures of (OV-AZO) and Its Metal Complexes

The synthesized Schiff base ligand (Ov-Azo) and its metal complexes were prepared under microwave irradiation and conventional reflux, as shown in Scheme 1. The Schiff base ligand and its metal complexes isolated under microwave irradiation conditions have the same physical properties (color, shape, melting point) compared with those synthesized by refluxing preparation. The comparison between yield and time of the preparation of the Schiff base ligand and its complexes are shown in Table 1. The results show that the synthesis of these compounds under microwave irradiation is faster and more productive and consumes less solvent than the conventional method, which verifies some green chemistry principles. The synthesized Schiff base ligand was soluble in methanol, acetone, acetonitrile, chloroform, DMF, and DMSO at room temperature and also soluble in hot ethanol. The prepared ligand and its metal complexes (1–6) are stable at room temperature. Metal complexes were synthesized by the stoichiometric reaction of the equivalent metal salts and the respective ligand in the molar ratio M:L of 1:1 for all complexes. Spectroscopic measurements and analytical data of complexes 1–6 are given in Tables 1–3. The potential sites (N and O) of the prepared Schiff base ligand coordinate with the metal ions of Mn(II), Co(II), Ni(II), Cu(II), Zn(II) and Zr(IV), producing metal complexes. Characterization of the prepared metal complexes approved the chelation mode of the ligand toward metals.

### 3.1 Infrared and NMR Spectra

**Figure 1:**
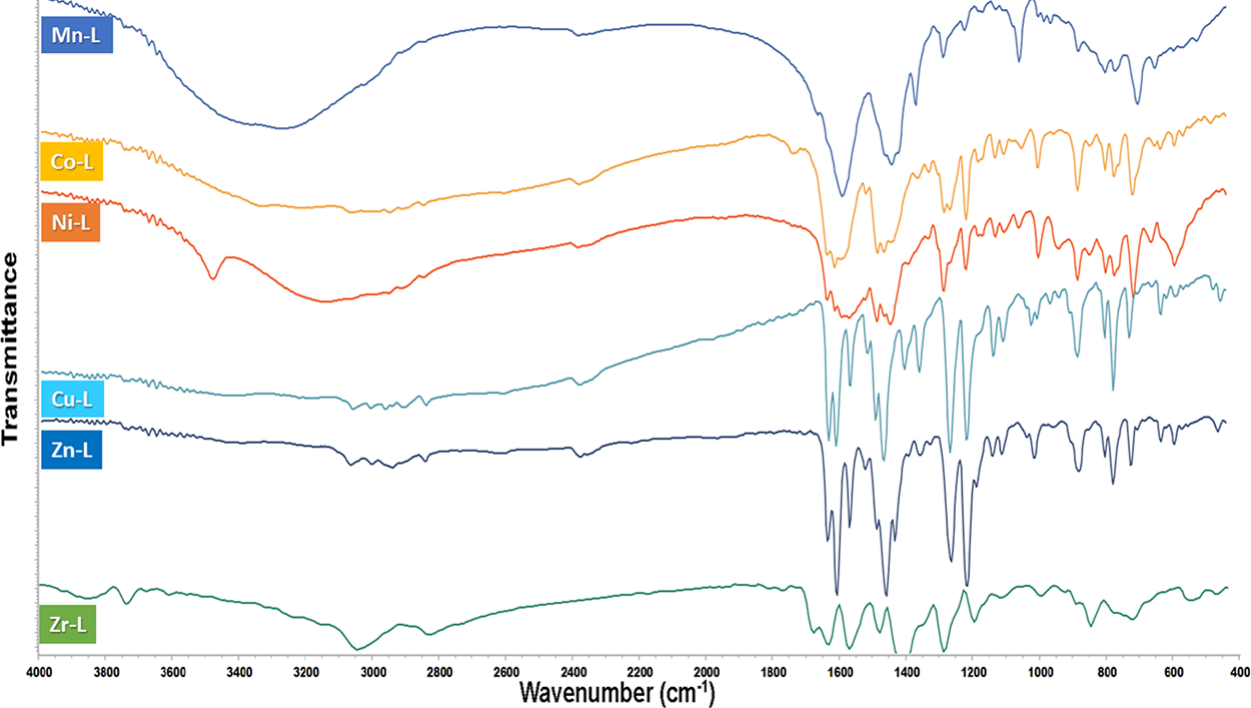
FTIR Spectrum of Mn(II), Co(II), Ni(II), Cu(II), Zn(II), and Zr(IV) complexes.

FTIR spectra of the Schiff base (OV-Azo) ligand and its metal complexes are presented in Figure S1 and Figure 1, respectively. The FTIR spectra of OV-Azo showed that the azomethine symmetric stretching frequency strong band at 1612 cm^–1^ is assigned to the imine, ν(C=N) group, and the phenolic ν(C–O) frequency was observed at 1261 cm^–1^. Moreover, the ligand shows a band at 1490 cm^–1^ due to the aromatic azo ν(N=N) group. The other bands at 2962, 3043, and 3421 cm^–1^ are assigned to ν(CH) aliphatic, ν(CH) aromatic, and ν(OH) H_2_O, respectively, as shown in Table 2. The mode of chelation of free Schiff base was suggested based on the comparison between its IR spectrum and spectra of the metal complexes.

**Table 2:** Characteristic FTIR (cm^–1^) of the Schiff Base Ligand and Its Metal Complexes

symbol	ν(OH)	ν(CH)_aromatic_ ν(CH)_aliphatic_	ν(C=N)	ν(N=N)	ν(OAC)	ν(Ar–O)	ν(M–O)	ν(M–N)	ν(M–Cl)
OV-Azo	3422	3043–2962	1612	1490		1261			
Mn-L (1)	3420	3063–2944	1610	1492	1416	1258	555	417	
Co-L (2)	3420	3054–2940	1610	1493	1416	1256	556	421	
Ni-L (3)	3468	3060–2940	1610	1490	1419	1257	556	421	
Cu-L (4)		3049–2952	1606	1490		1237	552	417	
Zn-L (5)		3058–2933	1610	1495		1235	556	425	
Zr-L (6)	3412	3038–2940	1612	1490		1255	563	424	409

The FTIR spectra of metal complexes reveal that the ligand binds to these metal ions in its bidentate, in which the coordination takes place through C=N and deprotonated OH, OAc, and H_2_O to metal ions. This strong evidence has been gathered based on the following: (i) the band due to azomethine (C=N) is shifted higher by 20–30 cm^–1^ of frequency in the spectra of all metal complexes. (ii) The coordination of the azomethine nitrogen is also confirmed by the appearance of new bands near 420 cm^–1^ assigned to the νM–N vibration.^27,39,40^ (iii) The band corresponding to νOH as a weak band may be due to the deprotonation of the OH, and the second of H_2_O. The coordination through oxygen is confirmed by the appearance of νM–O near 556 cm^–1^.^41^ (iv) The appearance of a band near 1405 cm^–1^ related to negative acetate ion ν(-OAC) is detected in Mn(II), Co(II), and Ni(II), suggesting the existence of carboxylic modes attached to those complexes. (v) The appearance of the band at 3422 cm^–1^, assigned to ν(OH) for complexes except for Cu(II) and Zn(II) highlights the absence of water from the mentioned complexes.^42^

Figure S2 shows the ^1^H NMR spectra of ligand (OV-Azo) in DMSO-*d*_6_. The chemical shifts of methoxy group protons (−OCH_3_) displayed a singlet peak at δ 3.84 ppm. The peaks at δ 2.5 and 3.36 ppm were attributed to DMSO and DMSO-H_2_O protons. A broad multiplet was observed at δ 6.91–8.0 ppm, attributed to aromatic protons. The chemical shifts (δ) of azomethine (—CH=), and phenolic (−OH) protons appeared at δ 9.04 and δ 12.94 ppm, respectively.^43^

### 3.2 UV–vis Spectra and Magnetic Susceptibility of Complexes

Electronic spectra and magnetic moment (the units are in B.M. (Bohr magnetons)) of the Schiff base ligand and its metal complexes Ni(II), Co(II), Zn(II), Cu(II), Mn(II), and Zr(IV) in DMSO are shown in Table 3. All these new compounds were scanned in the region of 14,286–52,631 cm^–1^ at concentrations between 50 μM and 1 mM at room temperature. The electronic data spectra of ligand (OV-AZO) exhibited three absorption bands at λ_max_ values of 35714, 28,170, and 26,737 cm^–1^, assigned to (π–π*, Phenyl), (*n*–π*, N=N),^44,45^ and (*n*–π* C=N) transitions, respectively. The electronic spectra of the Mn(II) complex exhibited bands at 37,037, 28,090, 27,027, 24,390 and 14,992 cm^–1^, corresponding to π–π*, (*n*–π*, N=N), (*n*–π*, C=N), 6A1g → 4T2g, and (Mn II → (πO) MLCT) transitions, respectively, suggesting an octahedral geometry around the Mn(II) ion with a magnetic moment value of 7.91 B.M.^21^ Electronic spectra for the Co(II) complex exhibited bands at 36630, 28090, 27,027, 24,331, and 17,123 cm^–1^, which referred to π–π*, (*n*–π*, N=N), (*n*–π*, C=N), 4A2g(F) → 4T2g(F), and 4A2g(F) → 4T1g(F) transitions, respectively, suggesting an octahedral geometry with a magnetic moment value of 6.17 B.M.^21,46^

**Table 3:** Magnetic Moment and Electronic Spectral Data of the Schiff Base Ligand and Its Metal Complexes

compound	λ_max_ cm^–1^	assignments	μ_eff_ (BM)	g_⊥_	g_∥_	suggested structure
OV-AZO	35,714	π–π*				
	28,170,	*n*–π*, N=N				
	26,737	*n*–π*, C=N				
Mn-L	37,037	π–π*	7.91			octahedral
	28,090	*n*–π*, N=N				
	27,027	*n*–π*, C=N				
	24,390	^6^A_1g_ → ^4^T_2g_				
	14,992	(Mn(II)→(πO) MLCT)				
Co-L	36,630	π–π*	6.17			octahedral
	28,090	*n*–π*, N=N				
	27,027	*n*–π*, C=N				
	24,331	^4^A_2g_(F) → ^4^T_2g_(F)				
	17,123	^4^A_2g_(F) → ^4^T_1g_(F)				
Ni-L	36,496	π–π*	4.78			tetrahedral
	27,397	*n*–π*, N=N				
	26,882	*n*–π*, C=N				
	24,938	LMCT				
	23,256	^1^A_1g_ → ^1^A_2g_				
Cu-L	35,714	π–π*	2.07	2.07	2.17	tetrahedral
	29,411	*n*–π*, N=N & *n*–π*,				
	28,327	N=N				
	26,737	*n*–π*, C=N				
	24,631	LMCT				
Zn-L	36,900	π–π*	di			octahedral
	28,090	*n*–π*, N=N				
	26,525	*n*–π*, C=N				
	16,978	d-d				
Zr-L	36,232	π–π*	di			trigonal bipyramidal
	26,385	*n*–π*, N=N				
	25,000	*n*–π*, C=N				
	24,331	LMCT				

**Figure 2:**
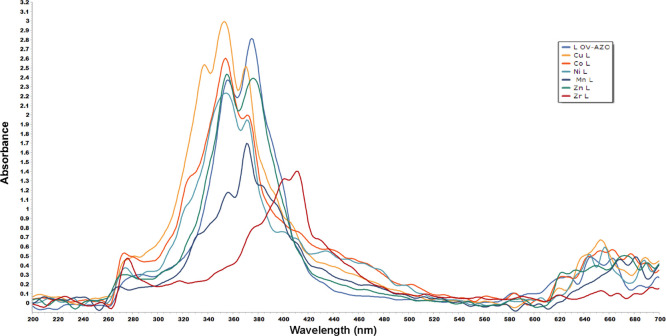
Schematic diagram of UV–Vis spectra of the Schiff base ligand and its complexes in DMSO.

The electronic spectra of the Ni(II) complex show bands of appreciable intensity at 36,496, 27,397, 26,882, 24,938, and 23,256 cm^–1^. These transitions have tentatively been assigned to π–π*, (*n*–π*, N=N), (*n*–π*, C=N), ligand to metal transfer (LMCT), and 1A1g → 1A2g transitions, respectively, suggesting a tetrahedral geometry with a magnetic moment value of 4.78 B.M.^47^ The electronic spectra for the Cu(II) complex shows bands at 35,714, 29,411, 28,327, 26,737, and 24,631 cm^–1^ , assigned to π–π*, (*n*–π*, N=N), (*n*–π*, N=N second ligand molecule), (*n*–π*, C=N), and LMCT transitions, respectively. The appearance of a new band at 29,411 cm^–1^ may be due to *n*–π*, N=N, revealing a second ligand molecule, indicating Cu(II) metal-binding with ligand in a 2:1 molar ratio; the magnetic moment is 2.07 B.M. Thus, the tetrahedral geometry has been suggested for the Cu(II) complex.^39,48^ The electronic spectra of the Zn(II) complex exhibited bands at 36,900, 28,090, 26,525, and 16,978 cm^–1^, assigned to π–π*, (*n*–π*, N=N), (*n*–π*, C=N), and d-d transitions, respectively, with diamagnetic properties, suggesting an octahedral geometry around the Zn(II) ion. For the Zr(IV) complex, it shows bands at 36,232, 26,385, 25,000, and 24,331 cm^–1^, attributed to π–π*, (*n*–π*, N=N), (*n*–π*, C=N), and LMCT transitions, respectively, with diamagnetic properties, suggesting a distorted trigonal bipyramidal geometry around Zr(IV).^49−51^ Comparative UV–vis spectra of the free Schiff base ligands (OV-Azo) and its metal complexes Ni(II), Co(II), Zn(II), Cu(II), Mn(II), and Zr(IV) in DMSO are shown in Figure 2, showing a match between the free Schiff base and different complexes.

### 3.3 Mass Spectra

**Figure 3:**
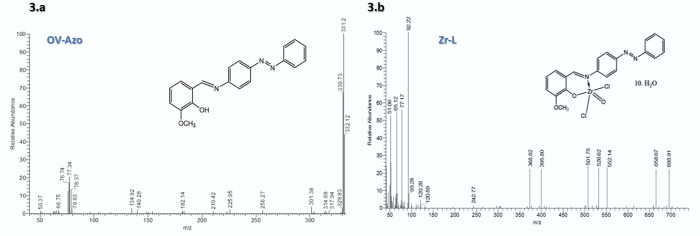
Mass spectra of Schiff base OV-Azo (a) and Zr-L (b) complex.

Figure 3a presents the mass spectrum of the ligand (OV-Azo). A molecular ion peak at *m*/*z* = 331.2 was found, confirming its structure (C_20_H_17_N_3_O_2_). The highest ion peak at *m*/*z* 330.7 is due to M^+^( C_20_H_16_N_3_O_2_), while the other characteristic peaks are observed at *m*/*z* values of 329, 317, 314, 301, 256, 225, 210, 182, 140,134,79 ,78, 77, 76, 66, and 50. Figure 3b shows the ion peak at *m*/*z* = 688 as the molecular peak of (C_20_H_36_C_l2_N_3_O_13_Zr). The highest ion peak at *m*/*z* 92 is due to M^+^( C_6_H_6_N^.^), while the other characteristic peaks were observed at *m*/*z* values of 658, 552, 526, 501, 395, 368, 242, 120, 93, 77, 65, and 51.

### 3.4 Electron Spin Resonance Spectra (ESR) Spectrum

**Figure 4:**
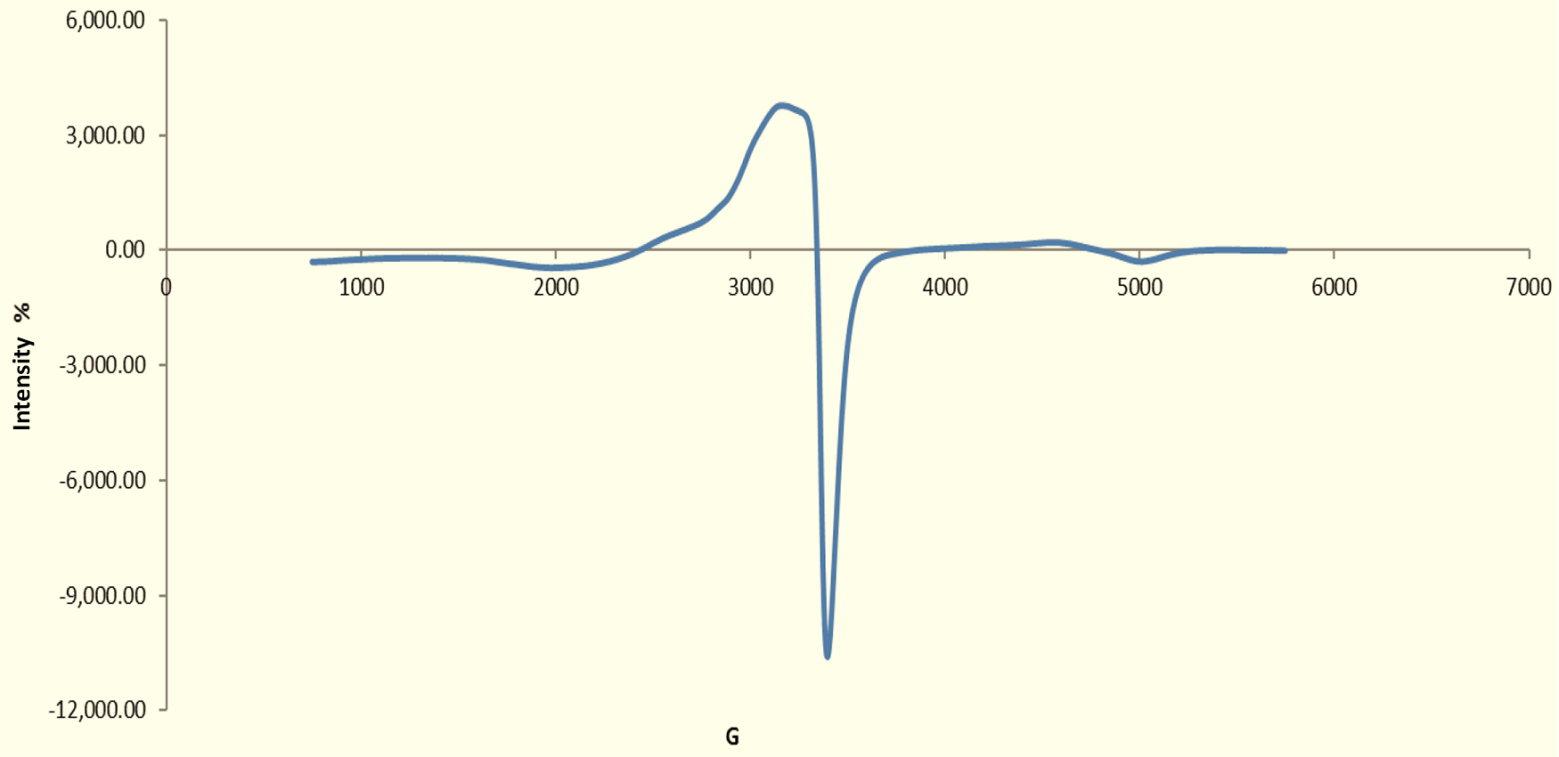
Schematic diagram of electron spin resonance (ESR) spectra of the Cu(II) complex.

Electron spin resonance spectra of the Cu(II) complex exhibited a broad signal with two ″*g*″ values (*g*_∥_, *g*_⊥_), as presented in Table 3 and Figure 4. The *g*_∥_ < *g*_⊥_ < 2.3 is characteristic of complexes with the 2B1(d*x*^2^ – *y*^2^) orbital ground state. The values of the *g* average were calculated by the following equation:
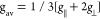
1

The Cu(II) complex exhibited *g*_∥_ value less than 2.3; this value signifies the covalent bond character between the copper metal and ligand, where *g*_∥_ < 2.3 concerns the ionic metal–ligand bond.^46^ A justly elevated value of *g* is compatible with the oxygen and nitrogen coordination in these complexes. This coupling is known as the hyperfine interaction. The electronic absorption spectra of the copper II complex indicated that it was formed in a tetrahedral structure.

### 3.5 Thermal Analysis and Thermo-Kinetic Parameters of Metal Complexes

**Figure 5:**
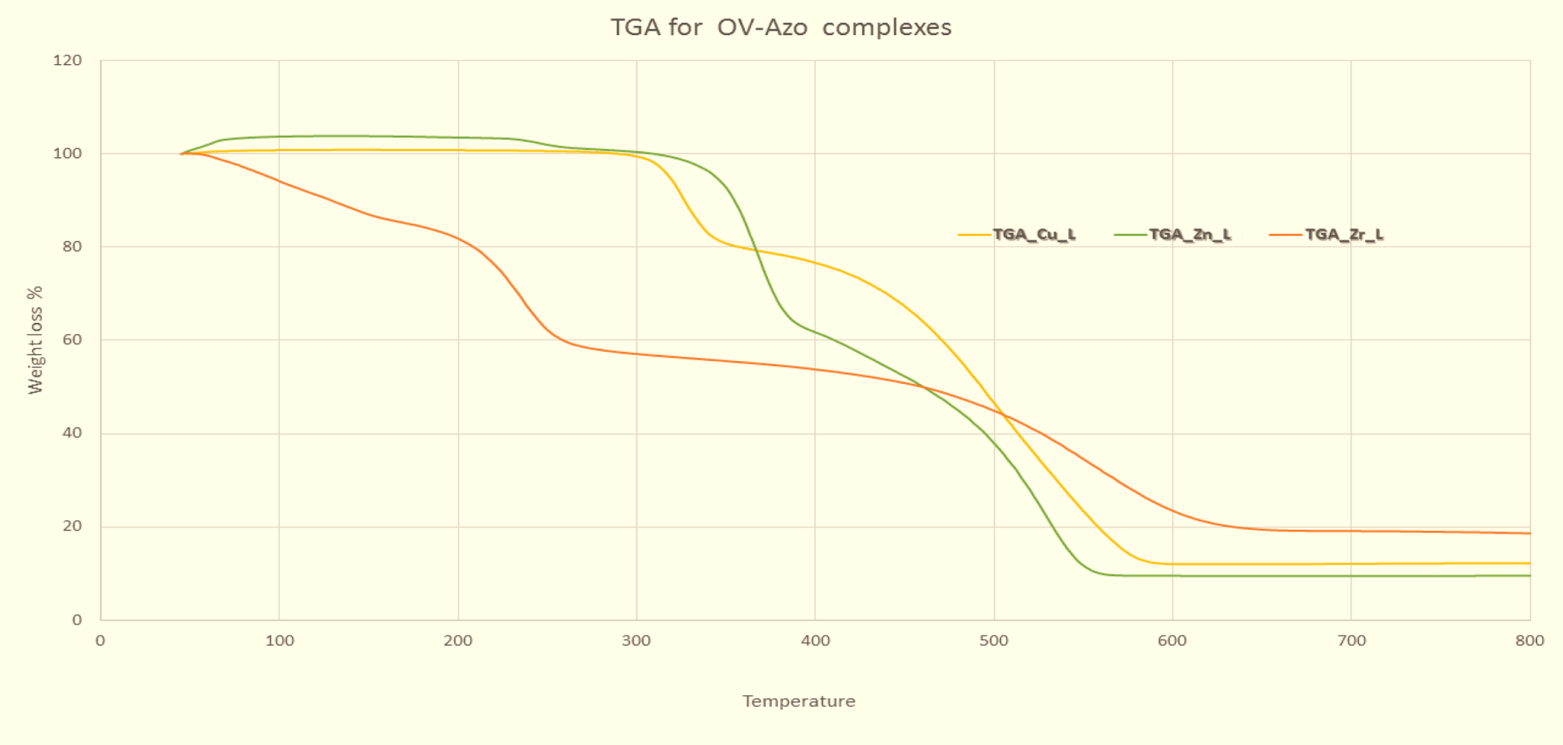
Thermographs of metal complexes of Cu, Zn, and Zr of OV-Azo.

**Scheme 2:**
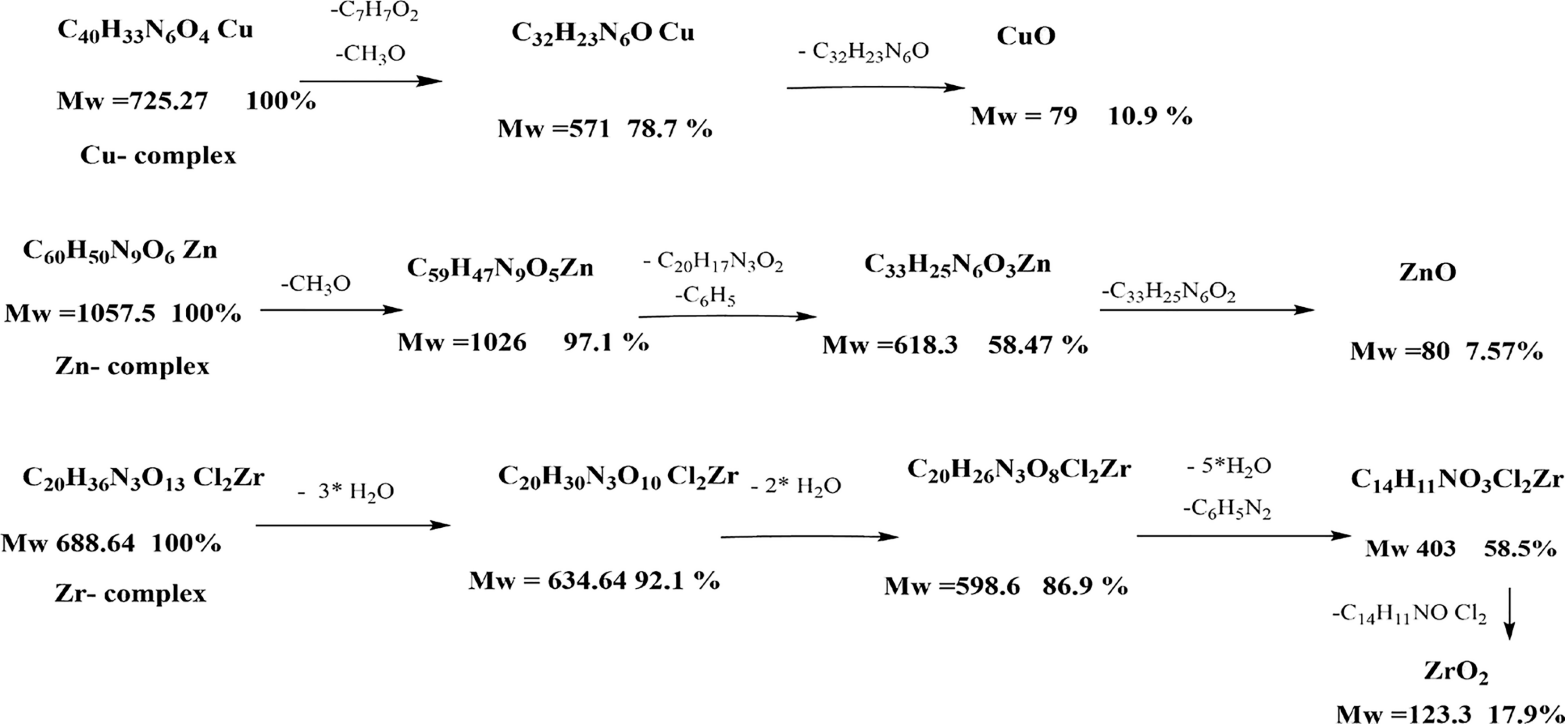
Sequence of Decomposition Steps for Cu(II), Zn(II), and Zr(IV) Complexes

In the present work, thermal analysis was carried out to get significant information about the thermal stability of the prepared complexes and to inspect the nature of solvent molecules (if existing) to be inside the inner coordination sphere of the metal or outside it.^52−54^ Thermal kinetic parameters of complex decomposition were evaluated using the Coats–Redfern relation^55^ and Horowitz–Metzger kinetic parameters.^56^ The entropy of activation (ΔS*), enthalpy of activation (ΔH*), and the free energy change of activation (ΔG*) were calculated. The obtained results were calculated throughout all stages for three complexes for each ligand using the Coats–Redfern and Horowitz–Metzger equations and shortened in Table 4. The thermogram of the Cu(II) complex showed two decomposition steps; the first step at a temperature range between 262 and 375 °C corresponds to the loss of organic parts with the molecular formula (C_7_H_7_O_2_) and (CH_3_O) (21.3%) molecule. The second step at a temperature range between 375 and 655 °C corresponded to the loss of organic parts with the molecular formula (C_13_H_10_N_3_) and (C_19_H_13_N_3_) (67.7%), leaving a residue of CuO (10.9%). The thermogram of the Zn(II) complex presents three decomposition steps; the first step at a temperature range between 186 and 292 °C represented the loss of organic part with the molecular formula (CH_3_O) (2.93%). The second step at a temperature range between 315 and 401 °C corresponded to the loss of organic parts with the molecular formula (C_6_H_5_) and (C_20_H_17_N_3_O_2_) (38.6%). The third step at temperature ranges between 401 and 617 °C revealed the loss of organic parts with the molecular formula (C_33_H_25_N_6_O_2_) (50.5%), leaving a residue of ZnO (7.57%). The thermogram of the Zr(IV) complex shows four decomposition steps; the first step at a temperature range between 44 and 118 °C corresponds to the loss of three molecules of water 3H_2_O (7.85%). The second step at a temperature range between 119 and 171 °C corresponds to the loss of two molecules of water 2H_2_O (5.23%). The third step at temperature ranges between 174 and 328 °C corresponds to the loss of five molecules of water 5H_2_O and an organic part with the molecular formula (C_6_H_5_N_2_) (28.3%). The last step at temperature ranges between 335 and 715 °C displayed the loss of organic parts with the molecular formula (C_14_H_11_ON) (40.5%), leaving (17.91%) ZrO_2_ as a residue. The sequence of decomposition steps for Cu(II), Zn(II), and Zr(IV) complexes are shown in Scheme 2, Figure 5, and Table 5.

**Table 4:** Thermodynamic Kinetic Parameters Data of the Thermal Decomposition of Complexes

		Coats–Redfern						Horowitz–Metzger					
compd no.	steps	*R*^2^	*E_a_* KJ mol^–1^	*A* S^–1^	ΔS* mol^–1^ K^–1^	ΔH* KJ mol^–1^	ΔG* KJ mol^–1^	*R*^2^	*E_a_* KJ mol^–1^	*A* S^–1^	ΔS* mol^–1^ K^–1^	ΔH* KJ mol^–1^	ΔG* KJ mol^–1^
Cu-L (4)	1st	0.99	434	2.2 × 10^30^	330	429	231	0.99	205	4.4 × 10^17^	78	200	153
	2nd	0.99	133	3.8 × 10^8^	–8	132	137	0.99	62	6.4 × 10^3^	–189	56	189
Zn-L (5)	1st	0.96	85	5.6 × 10^9^	–63	81	112	0.98	44	8.5 × 10^3^	–184	40	132
	2nd	0.99	531	1.1 × 10^36^	192	526	406	0.99	230	1.6 × 10^19^	105	224	159
	3rd	0.98	423	6.6 × 10^21^	33	417	392	0.99	203	5.7 × 10^13^	6	196	192
Zr-L (6)	1st	0.99	165	1.5 × 10^17^	82	162	132	0.99	79	2 × 10^11^	–36	76	89
	2nd	0.99	324	3.4 × 10^34^	100	321	278	0.99	154	8.1 × 10^18^	101	150	107
	3rd	0.99	263	1.3 × 10^20^	–23	258	271	0.99	131	2.5 × 10^12^	–19	126	137
	4th	0.99	148	2 × 10^9^	–144	142	243	0.99	72	3.9 × 10^4^	–174	66	188

**Table 5:** Thermo-Gravimetric TGA Data of some Selected Complexes

				Δ*T* °C		mass %		
compound	molecular formula	Mwt	steps	*T*_i_	*T*_f_	calcd	found	assignment
Cu-L (4)	C_40_H_33_N_6_O_4_ Cu	725.27	1st	262	375	21.3	21.8	C_7_H_7_O_2_, CH_3_O
			2nd	375	655	67.7	66.49	C_13_H_10_N_3_, C_19_H_13_N_3_
			residue			10.9	11.61	CuO
Zn-L (5)	C_60_H_50_N_9_O_6_Zn	1057.5	1st	186	292	2.93	2.95	CH_3_O
			2nd	315	401	38.6	37.52	C_6_H_5_, C_20_H_17_N_3_O_2_
			3rd	401	617	50.3	51.31	C_33_H_25_N_6_O_2_
			residue			7.57	7.42	ZnO
Zr-L (6)	C_20_H_36_N_3_O_13_Cl_2_ Zr	688.64	1st	44	118	7.85	8.5	3*H_2_O
			2nd	119	171	5.23	6.29	2*H_2_O
			3rd	174	328	28.3	28.67	5*H_2_O, C_6_H_5_N_2_
			4th	335	715	40.5	36.95	C_14_H_11_NO Cl_2_
			residue			17.91	18.99	ZrO_2_

### 3.6 Scanning Electron Microscope (SEM)

**Figure 6:**
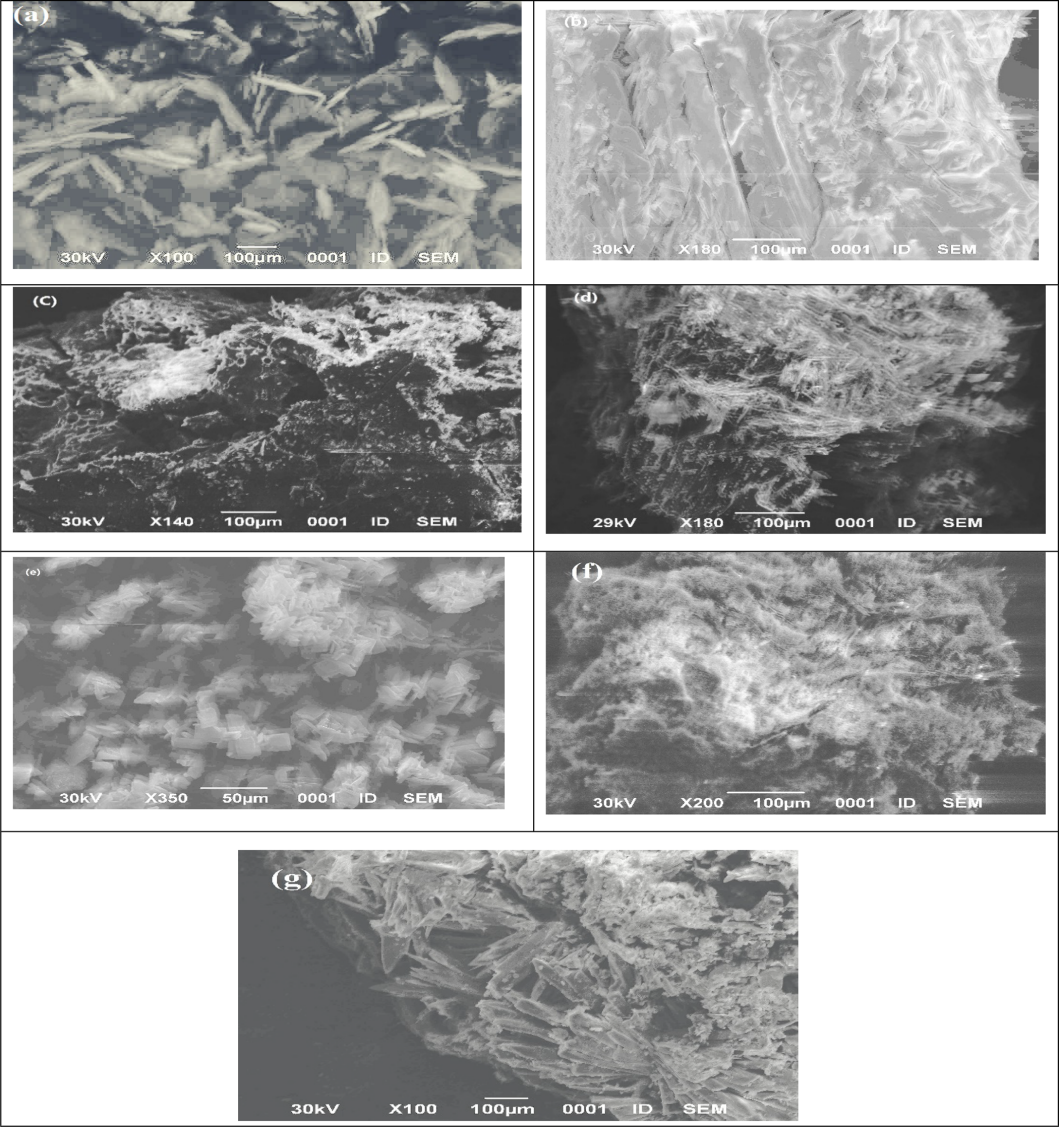
SEM images of ligand OV-AZO and its complexes: (a) Schiff base, (b) Mn, (c) Co, (d) Ni, (e) Cu, (f) Zn, and (g) Zr.

The surface morphology of the free Schiff base ligand OV-Azo and its complexes is shown in Figure 6. The surface morphology of the complexes Ni(II), Co(II), Zn(II), Cu(II), Mn(II), and Zr(IV) indicates the existence of crystals free from any shadow of the metal ion on their external surface (Figure 6b–g). These SEM images were quite different compared to the free Schiff base ligand.

### 3.7 Particle Size Distribution

**Figure 7:**
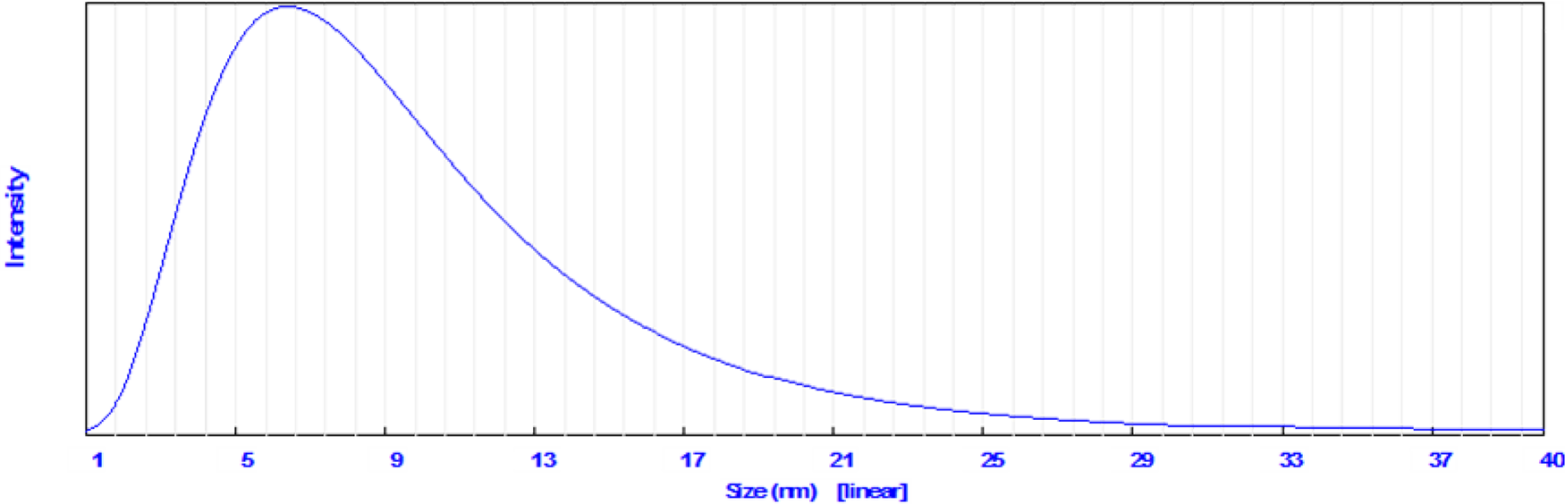
Particle size distribution of the prepared Cu(II) complex at a diffraction angle of 10.9°.

The particle size of the newly prepared complexes under microwave irradiation was measured using a particle size analyzer at a diffraction angle of 10.9°. The results show that the mean particle size of Co(II), Zn(II), Cu(II), Mn(II), and Zr(IV) complexes are 17.7, 28.9, 7.1, 26.7, and 37.5 nm, respectively, while the particle size of Ni(II) is 581 nm. For example, Figure 7 shows the schematic diagram of the particle size distribution of the Cu(II) complex. These results support the use of microwave irradiation to synthesize nanoparticles in comparison with conventional conditions.^57^

## 4 Biological Activity

### 4.1 Antimicrobial Activity

**Figure 8:**
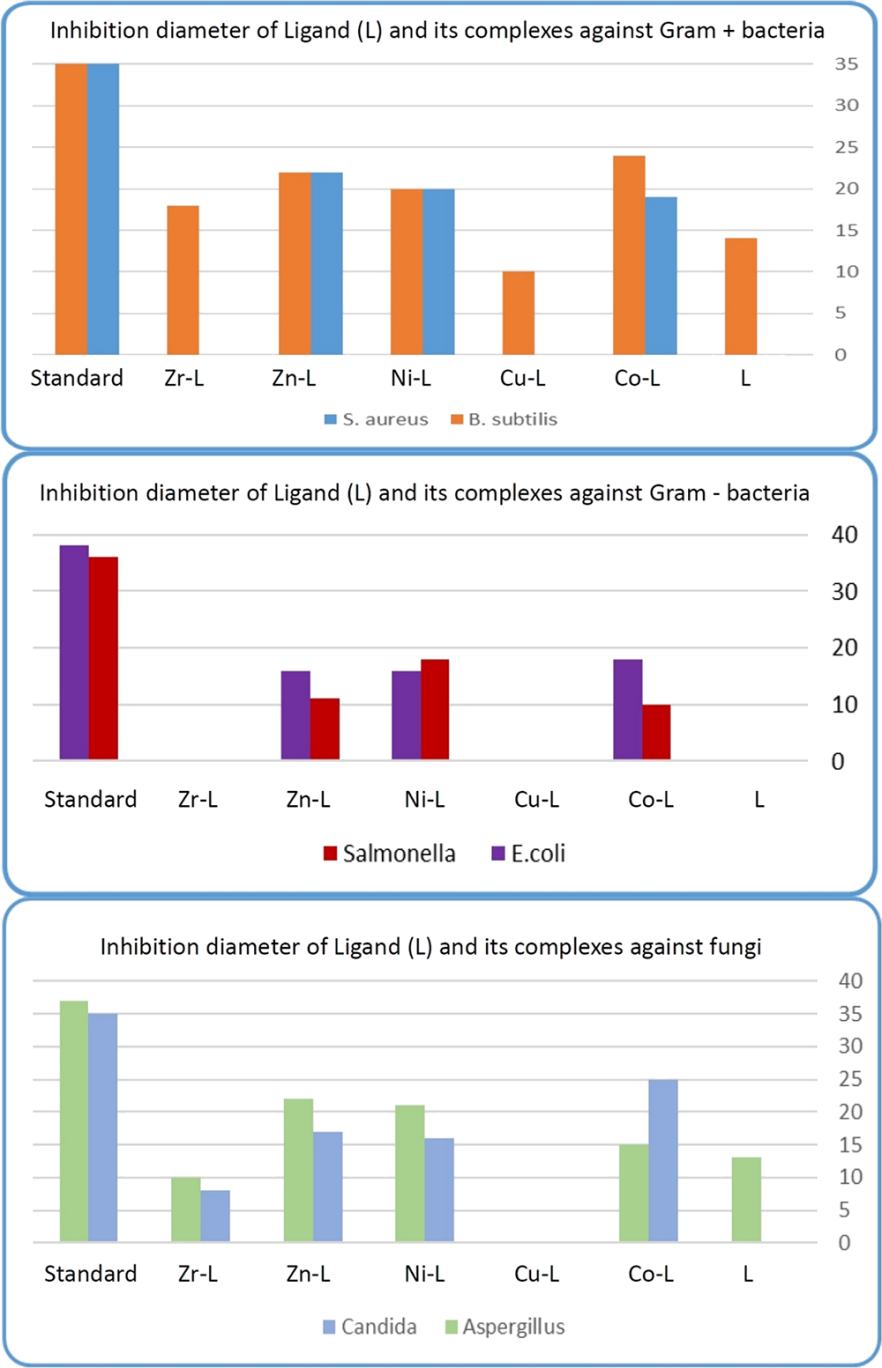
Antimicrobial activity of the Schiff base ligand and its metal complexes against different strains.

Results of the antibacterial and antifungal activities of the isolated Schiff base ligand and their metal complexes are presented in Table 6. The antimicrobial activity taken as the inhibition zone diameter is depicted graphically in Figure 8. The free Schiff base ligand (OV-Azo) is biologically inactive, and its metal complexes, more active upon chelation, are qualified for Tweedy’s chelation theory.

**Table 6:** Antimicrobial Result of the OV-Azo Ligand and Its Related Metal Complexes

	Gram-positive bacteria		Gram-negative bacteria		yeasts and fungi	
sample code	*Staphylococcus aureus* (ATCC 25923)	*Bacillus subtilis* (ATCC 6635)	*Salmonella typhimurium* (ATCC14028)	*Escherichia coli* (ATCC25922)	*Candida albicans* (ATCC10231)	Aspergillus fumigatus**
L	NA	14	NA	NA	NA	13
Co-L	19	24	10	18	25	15
Cu-L	NA	10	NA	NA	NA	NA
Ni-L	20	20	18	16	16	21
Zn-L	22	22	11	16	17	22
Zr-L	NA	18	NA	NA	8	10
control	35	35	36	38	35	37
DMSO	0	0	0	0	0	0

The chelation process decreases the metal atom polarity mainly because of the positive charge of the metal partially shared with nitrogen and oxygen atoms present on the free Schiff base ligand, and there is electron delocalization over the whole complex ring. This increases the lipophilic character of the metal chelate and favors its permeation through the lipid layers of the microbial membranes.

From Table 6, the low activity of some metal complexes is observed. This poor inhibition performance may be attributed to the low lipophilicity, which decreases the ability of the metal complex to penetrate the lipid membrane^.^^58^ Also, the presence of the metal ions in complexes with azomethine derivatives exhibited more effectiveness in membrane destabilization than the free ligand. This leads to disturbing the structural integrity of the cell and hence eradicating the microorganism.^59^ Complexes of Zn(II), Co(II), and Ni(II) complexes of OV-Azo showed a moderate to high inhibition zone diameter and are bactericidal against Gram-positive bacteria (*Staphylococcus aureus* and *Bacillus subtilis*). Furthermore, they achieved a moderate to high inhibition zone diameter and antifungal activity against fungus (*Candida albicans* and *Aspergillus fumigatus*).

### 4.2 Anticancer Activity

The metal complexes are considered a promising core for designing a new cancer drugs with low IC_50_ and low side effects.^60^ The data is depicted in Table 7 and Figures S3 and S4. It was found that the Schiff base ligand and its Cu(II), Zr(IV), and Mn(II) complexes exhibited the highest cytotoxic activity against HepG-2 and HCT as compared to Cisplatin as a reference drug. This might be attributed to lower concentrations of the ligand and its complexes sufficient to decrease the viability of these cancer cells. It was noticed that the Cu(II) complex showed the highest cytotoxic activity (IC_50_ 18 and 22 μg/mL for HepG-2 and HCT, respectively) more than the ligand itself. This was followed by Mn (IC_50_ 20 and 24 μg/mL for HepG-2 and HCT, respectively) and Zr-ligand complexes (IC_50_ 22 and 27 μg/mL for HepG-2 and HCT, respectively). On the contrary, Co, Zn, and Ni-ligand complexes showed lower cytotoxic activity. During the current study, it was found that the metal complexes can inhibit the growth of cancer cells at convergent concentrations. This was in agreement with Skladanowski et al.^61^ and was supported recently by Qin et al.^62^

**Table 7:** Cytotoxic Activity of OV-Azo Ligand and its Related Metal Complexes against Human Liver cancer Cells (HepG-2) and Colon Carcinoma (HCT-116)

	median inhibitory concentration (IC_50_) in μg/mL							
cell line	L	Mn-L (1)	Co-L (2)	Ni-L (3)	Cu-L (4)	Zn-L (5)	Zr-L (6)	cisplatin
HepG-2	24	20	42	35	18	39	20	8.4
HCT-116	28	24	50	48	22	45	27	6.92

It was also reported that metal complexes possess higher cytotoxic activity against human cancer cells.^62^ There are numerous potential mechanisms of anticancer activity of metal complexes. This might refer to the ability of these metal complexes to induce S-phase arrest in cancer cells associated with increased expression of tumor suppressors’ genes.^63^ The activity of the metal complexes is the binding not only with DNA but also with proteins and should be analyzed as the targeted molecules in anticancer mechanisms.^36^ The Cu(II) complex exhibited the highest cytotoxic activity by a well-known mechanism of action through DNA interaction and cleavage, in which DNA is degraded by a Fenton-type reaction.^64,65^ Also, it targets the nucleic acids directly by the cleavage of DNA and RNA through the displacement of other metal ions.^66^ Furthermore, the newly synthesized Cu complex caused oxidative stress. This led to cell death in cancer cells by increasing the total oxidant status associated with decreasing total antioxidant levels, raising the oxidative stress level. This is also a reliable indicator signifying the oxidant–antioxidant balance by evaluating total antioxidant and oxidant status.^67,68^ In addition, the Cu–ligand complexes were presented to activate oxygen species and inhibit the growth of Ehrlich ascites tumor cells.^69^

## 5 Conclusions

New metal complexes of Ni(II), Co(II), Zn(II), Cu(II), Mn(II), and Zr(IV) incorporating Schiff base were synthesized using microwave-assisted irradiation compared to conventional conditions. The thermal dehydration and decomposition of Mn(II), Ni(II), and Zn(II) complexes show dehydration of water and elimination of acetate. Organic content and metal oxide MO remained as a residue. The antimicrobial activity of the ligand and its metal complexes against the bacterial and fungal strains exhibited that Schiff base ligand is biologically inactive, and its metal complexes that are more active upon chelation is attributed to Tweedy’s chelation theory. Complexes of Co(II), Ni(II), and Zn(II) of OV-Azo achieved a moderate to high inhibition zone diameter and antimicrobial activity against bacteria and fungus. Schiff base ligand and its Cu(II), Zr(IV), and Mn(II) complexes exhibited the highest cytotoxic activity against HepG-2 and HCT compared to cisplatin as a reference drug. The results evidently exhibit that the entry of metals into organic compounds enhances their efficiency in destroying cancer cells especially copper and manganese ions; this makes us focus on these metal ions with organic substances that have higher biological activity in subsequent studies.

## Supporting Information Available

(Figure S1) FTIR spectrum of Schiff base ligand (OV-Azo), (Figure S2) ^1^H NMR spectra of Schiff base ligand (OV-Azo), (Figure S3) cytotoxic activity against human liver cancer cells (HepG-2), and (Figure S4) cytotoxic activity against human colon carcinoma (HCT) (PDF)
