# Supplementary material for: Green Synthesis, Characterization, Antimicrobial and Anticancer Screening of New Metal Complexes Incorporating Schiff Base
Source: ACS Omega. 2022 Aug 26;7(36):32418–31. doi: 10.1021/acsomega.2c03911 (PMC9475620; doi:10.1021/acsomega.2c03911)
Supplement: Supplementary file 1 — ao2c03911_si_001.pdf [file ao2c03911_si_001.pdf]

Supporting Information

Green synthesis, Characterization, Antimicrobial and Anticancer Screening of New Metal Complexes Incorporating Schiff Base

Ali M. Hassan[1], Ahmed O. Said[2], Bassem H. Heakal[3], Ahmed Younis[4,*] Wael M. Aboulthana[5], and Mohamed F. Mady[4,6*]

[1] Chemistry Department, Faculty of Science, Al-Azhar University, Nasr City 11884, Egypt

[2] Senior researcher chemist, Greater Cairo Water Company, Cairo, Egypt.

[3] Research Laboratory, Cairo Oil Refining Company, Mostorod, Kaliobia, Egypt.

[4]Department of Green Chemistry, National Research Centre, Cairo 12622, Egypt.

[5]Biochemistry Department, Genetic Engineering and Biotechnology Research Division, National Research Centre, 33 El Bohouth St. (former EL Tahrir st.)-Dokki, Giza, Egypt-P.O.12622.

[6]Department of Chemistry, Bioscience and Environmental Engineering, Faculty of Science and Technology, University of Stavanger, N-4036 Stavanger, Norway.

**Figure S1:** FTIR Spectrum of Schiff base ligand (OV-Azo).	S2
**Figure S2.** ^{1}H NMR spectra of Schiff base ligand (OV-Azo).	S2
**Figure S3.** Cytotoxic activity against human liver cancer cells (HepG-2).	S3-S4
**Figure S4.** Cytotoxic activity against human colon carcinoma (HCT).	S3-S4
**Figure S5.** Mass fragmentation of Zr (IV) complex of ligand OV-AZO (L3)	S4

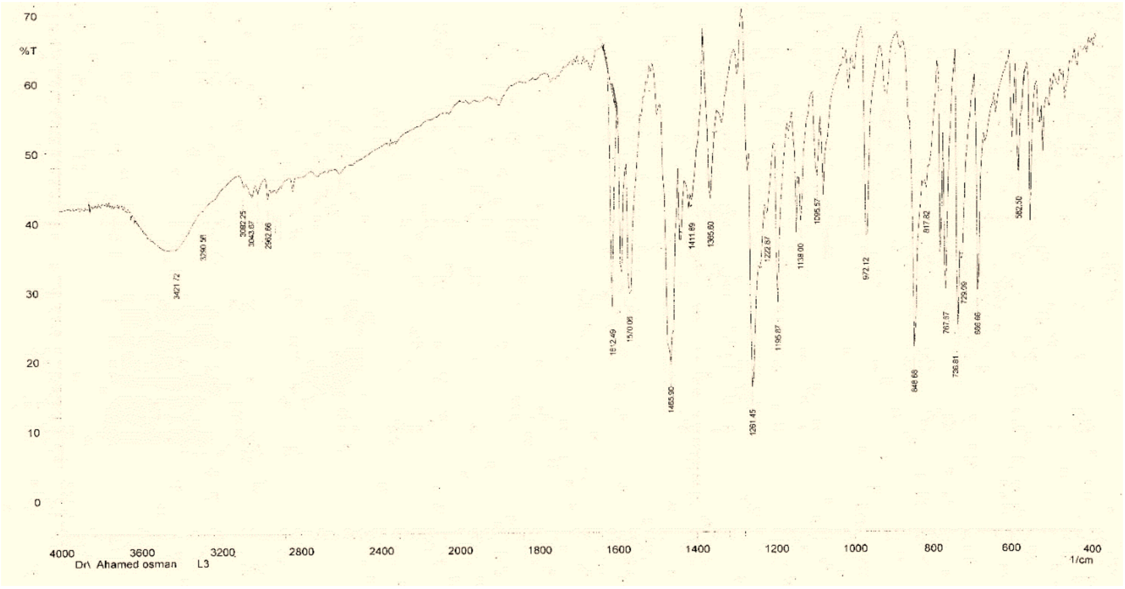

Figure S1: FTIR Spectrum of Schiff base ligand (OV-Azo).

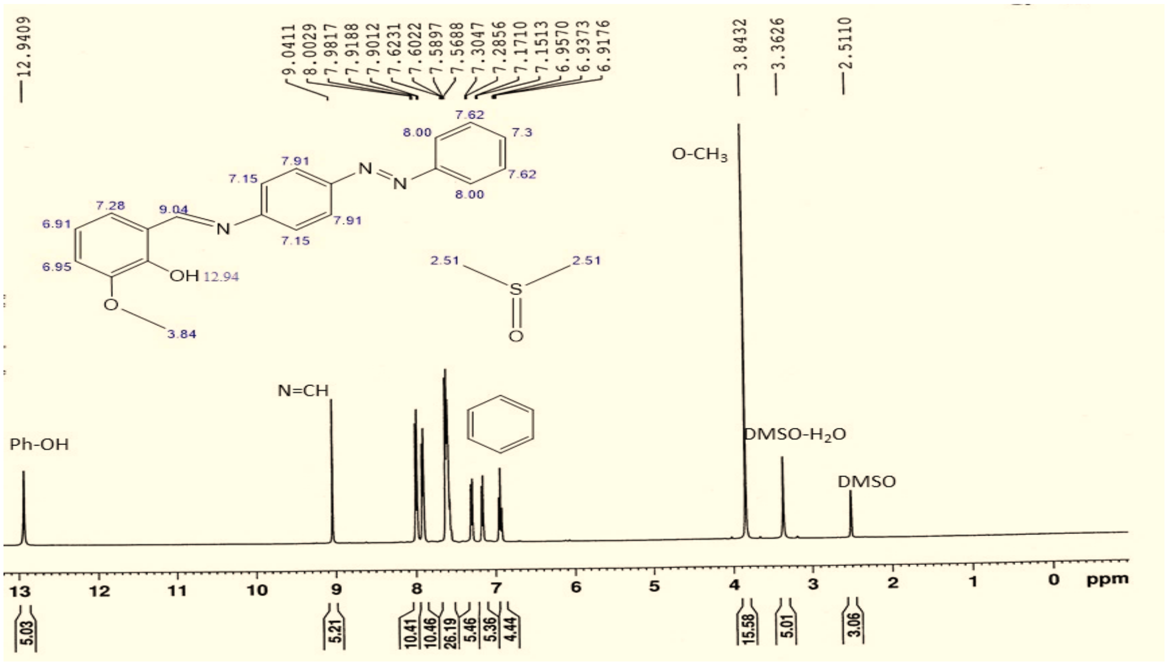

Figure S2. ^{1}H NMR spectra of Schiff base ligand (OV-Azo).

<table>
<tr><td>Figure S3. Cytotoxic activity against human liver cancer cells (HepG-2).</td><td></td><td>Figure S4. Cytotoxic activity against human colon carcinoma (HCT).</td></tr>
<tr><td>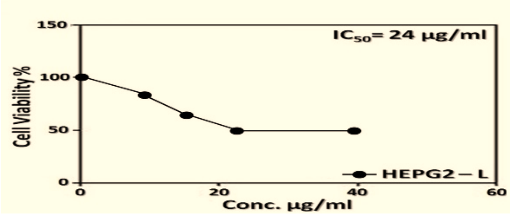
</td><td>L (OV-Azo)</td><td>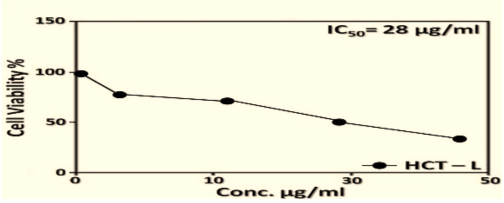
</td></tr>
<tr><td>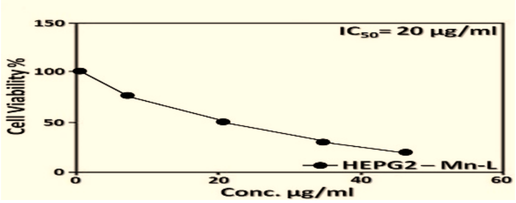
</td><td>Mn-L (1)</td><td>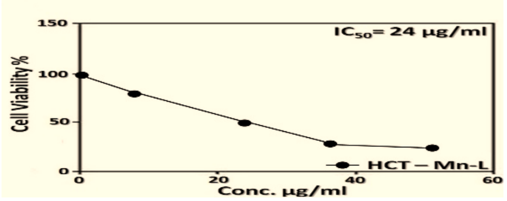
</td></tr>
<tr><td>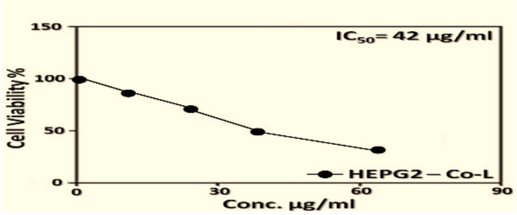
</td><td>Co-L (2)</td><td>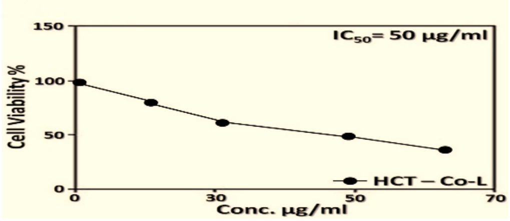
</td></tr>
<tr><td>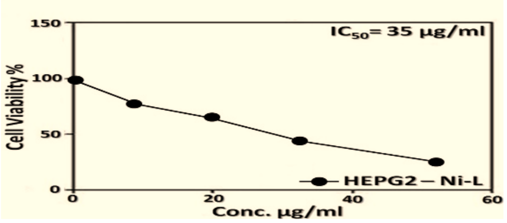
</td><td>Ni-L (3)</td><td>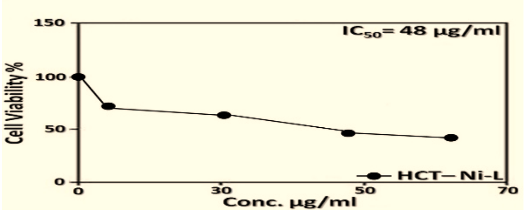
</td></tr>
<tr><td>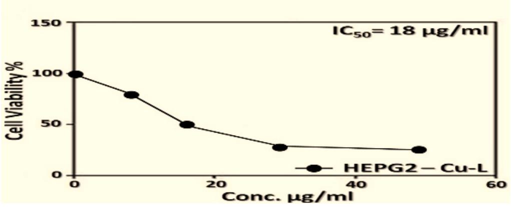
</td><td>Cu-L (4)</td><td>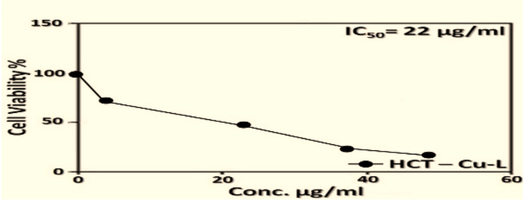
</td></tr>
<tr><td>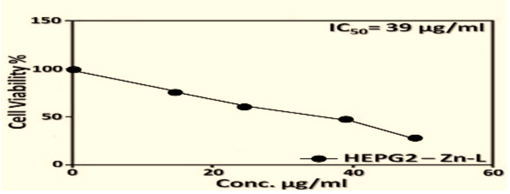
</td><td>Zn-L (5)</td><td>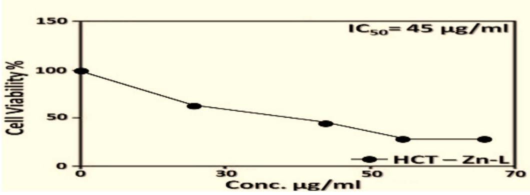
</td></tr>
</table>

<table>
<tr>
<td>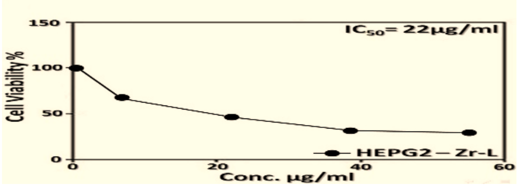
</td>
<td>Zr-L (6)</td>
<td>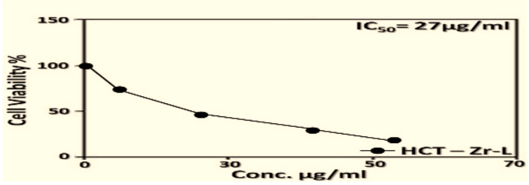
</td>
</tr>
</table>

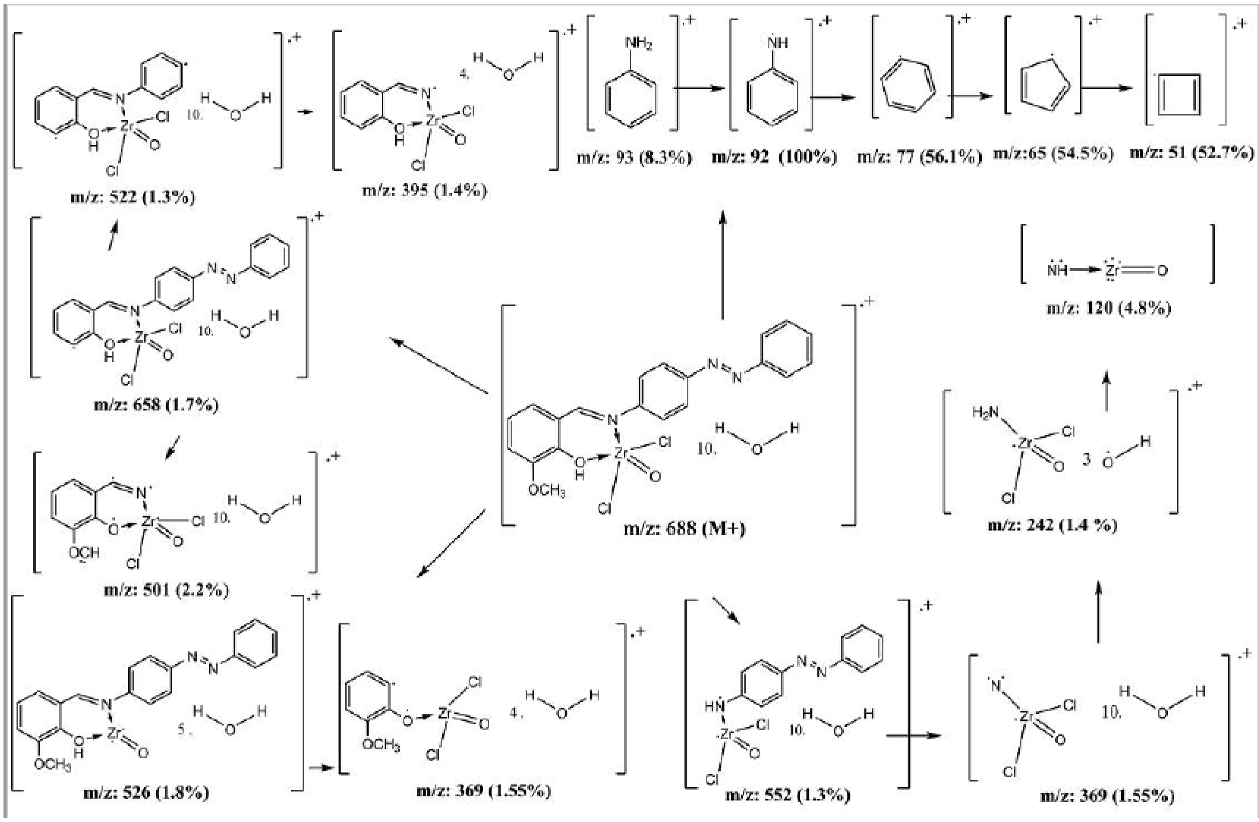

Figure S5. Mass fragmentation of Zr (IV) complex of ligand OV-AZO (L3)
